# Intervention for Psychological Trauma in Children Impacted by War in Ukraine

**DOI:** 10.1001/jamanetworkopen.2025.3057

**Published:** 2025-03-14

**Authors:** Irwin Redlener, Khrystyna Dudashvili, Oksana Viontsek, Roy Grant, Eva Roca

**Affiliations:** 1 National Center for Disaster Preparedness, Columbia University, New York, New York; 2 Ukraine Children’s Action Project (UCAP), Los Angeles, California; 3 Recovery Camp, Lviv, Ukraine

## Abstract

This cohort study describes outcomes of a 6-day psychosocial recovery camp as an intervention for psychological trauma experienced by children from front-line regions of the war in Ukraine.

## Introduction

The war in Ukraine escalated in February 2022 with Russia’s full-scale invasion. The impact on Ukraine’s civilian population and infrastructure is devastating. Children bear a disproportionate burden,^1^ facing recurrent trauma, long-term displacement, and disruption or destruction of homes, schools, and other supports.^2^ To address this burden, 6-day psychosocial recovery camps using a mother-child intervention model were conducted from September 2022 to July 2024 in Lviv, Ukraine. The camps had 4 goals: providing safety away from armed conflict; screening children at risk for posttraumatic stress disorder (PTSD), with referrals for evaluation and treatment; presenting and reinforcing stress management skills; and arranging ongoing support and follow-up. We describe the intervention and its preliminary results among children from front-line regions in Ukraine.

## Methods

Each age-appropriate, 6-day camp comprised 75 participants, including children and their mothers, and was staffed by 6 stress management and recreational counselors and 3 mental health professionals with master’s degrees. Activities included play therapy, art therapy, music, dance, breathing techniques, and yoga. A cognitive approach was used when traumatic material was presented. Mothers participated in stress management support and information groups. Recreational counselors received ongoing psychotherapy training and coordinated care with mental health professionals. Participants were recruited from front-line regions (Odessa, Kharkiv, and Donetsk) through family service organizations, social media, and a message application (Telegram). Written consent was obtained to disseminate anonymized information. This cohort study was approved by the Ukrainian Institute on Public Health Policy Institutional Review Board. We followed the STROBE reporting guideline. Data were analyzed with descriptive statistics using Stata 15 (StataCorp LLC).

Intake included registration, parent interview, and administration of the Child and Adolescent Trauma Screen 2 (CATS-2). The CATS-2 identified exposure to stressful events, PTSD symptoms consistent with *Diagnostic and Statistical Manual of Mental Disorders* (Fifth Edition) and *International Classification of Diseases, 11th Revision* criteria experienced during the past 4 weeks (reported on a scale of 0 [never] to 3 [almost always]), and functional impairment. Symptom scores were categorized as no trauma-related distress, moderate distress, elevated distress, or high distress with probable PTSD. This instrument has strong reliability^3^ and was validated for use during the war in Ukraine.^4,5^ The CATS-2 was administered in Ukrainian using age-appropriate caregiver-report forms. Self-report forms were filled out by 60 adolescents.

At the conclusion of camp, each mother had an unstructured exit interview in which they discussed concerns and changes they observed in their children. Stress management skills were reinforced. Children needing further intervention were referred to mental health professionals. Online support groups for mothers were arranged. A rehabilitation specialist was included in each group, and a mental health professional was available as needed. One week after the end of camp, telephone follow-up to facilitate referrals was initiated. Outcomes were analyzed with descriptive statistics.

## Results

The sample included 1291 children (mean age 9.3 [range, 6 to 16] years; 633 females [49.0%]) and their mothers (963). Of the children, 438 (33.9%) had CATS-2 scores indicating elevated distress or probable PTSD associated with traumatic experiences (Table 1). The most frequently reported symptoms, occurring almost always, were difficulty concentrating, nervousness or restlessness, and sleep problems.

**Table 1.** Traumatic Experiences of Children Reported at Camp Intake

Experience	No. (%) (N = 1291)^a^
Lived within 30 km (18 miles) of hostilities	644 (49.9)
Internally displaced persons	891 (69.0)
Had a close relative in the war	300 (23.2)
Lost a close relative in the war	108 (8.4)
Lost their home	430 (33.3)

^a^ Children could have more than 1 of the traumatic experiences listed.

Following camp participation, 436 mothers (2 lost to follow-up) were contacted by staff to verify that they had referral information. Of these, 346 (79.4%) contacted or planned to contact a mental health resource; 233 (53.4%) were referred to a mental health professional. Despite the brevity of the intervention, many children (968 [75.0%]) had an overall improvement in psychosocial state after the intervention (Table 2).

**Table 2.** Changes in Children Reported Following Intervention

Change	No. (%) (N = 1291)^a^
Overall psychosocial state improved	968 (75.0)
Able to enjoy activities again	891 (69.0)
Less dependent on gadgets	852 (66.0)
More physical activity	775 (60.0)
Better communication with other children	710 (55.0)
More hugs	529 (41.0)
Less isolated	426 (33.0)
Sleep improved	232 (18.0)

^a^ Children could have more than 1 reported change.

## Discussion

This intervention is a replicable model, providing temporary respite from war exposure. It was associated with short-term improvement in children’s psychosocial state. The mental health referral rate compares favorably to rates in high income countries (43%).^6^ Limitations include lack of generalizability due to cohort composition (ie, children from front-line regions) and lack of preintervention and postintervention outcome assessments owing to the brief intervention. To address the finding that two-thirds of the children did not show symptoms indicating elevated distress or probable PTSD, intake interviews have been modified to explore risk and resilience factors that potentially modulate the impact of war on children.

## Notes

Supplement.
Data Sharing Statement
