# Error in the Author Affiliations

**DOI:** 10.1001/jamanetworkopen.2025.11438

**Published:** 2025-04-15

In the Research Letter titled “Intervention for Psychological Trauma in Children Impacted by War in Ukraine,”^1^ published online March 14, 2025, Mr Grant’s affiliation was incorrect. The correct affiliation is Recovery Camp, Lviv, Ukraine. This article has been corrected.
