# Supplementary material for: Intervention for Psychological Trauma in Children Impacted by War in Ukraine
Source: JAMA Netw Open. 2025 Mar 14;8(3):e253057. doi: 10.1001/jamanetworkopen.2025.3057 (PMC11909603; doi:10.1001/jamanetworkopen.2025.3057)
Supplement: Supplement. — Data Sharing Statement [file jamanetwopen-e253057-s001.pdf]

Data Sharing Statement

Redlener. Intervention for Psychological Trauma in Children Impacted by War in the Ukraine. *JAMA Netw Open*. Published April 03, 2025. doi:10.1001/jamanetworkopen.2025.3057

Data
Data available: No
